# Food Consumption during Pregnancy and Post-Partum. ECLIPSES Study

**DOI:** 10.3390/nu11102447

**Published:** 2019-10-14

**Authors:** Cristina Jardí, Estefania Aparicio, Cristina Bedmar, Núria Aranda, Susana Abajo, Gemma March, Josep Basora, Victoria Arija, the ECLIPSES Study Group

**Affiliations:** 1 Nutrition and Public Health Unit, Research Group on Nutrition and Mental Health (NUTRISAM), Faculty of Medicine and Health Science, Universitat Rovira i Virgili, 43201 Reus, Spain; cristina.jardi@urv.cat (C.J.); estefania.aparicio@urv.cat (E.A.); cristina.bedmar@urv.cat (C.B.); nuria.aranda@urv.cat (N.A.); 2 Pere Virgili Institute for Health Research (IISPV), Universitat Rovira i Virgili, 43003 Tarragona, Spain; 3 Sexual and Reproductive Healthcare Service of Reus-Tarragona, Institut Català de la Salut, Generalitat de Catalunya, 43003 Tarragona, Spaingmarch.tarte.ics@gencat.cat (G.M.); 4 Tarragona-Reus Research Support Unit, Jordi Gol Primary Care Research Institute, 43003 Tarragona, Spain; jbasora.tarte.ics@gencat.cat; 5 CIBERobn (Center for Biomedical Research in Physiopathology of Obesity and Nutrition), Instituto de Salud Carlos III, 28029 Madrid, Spain

**Keywords:** pregnancy, lactation, post-partum, food consumption, maternal factors, Mediterranean diet

## Abstract

Inadequate maternal diet can adversely affect mother and child. Our aim was to assess adherence to the Spanish dietary guidelines and to the Mediterranean diet, to analyze changes in diet during pregnancy and post-partum, and to identify maternal factors associated with food consumption. A total of 793 healthy pregnant women were recruited during the first prenatal visit and followed until the post-partum period. Data from the clinical history, anthropometric measurements, and lifestyle habits were collected. Food consumption was evaluated using a food frequency questionnaire. The results show that in pregnant women the consumption of healthy foods did not meet recommendations, whereas consumption of red and processed meat and sweet food exceeded recommendations. The results also show a medium adherence to the Mediterranean diet that remained unchanged throughout pregnancy. A significant decrease was observed in the consumption of fruits, followed by vegetables and then salted and sweet cereals from pregnancy to post-partum. A better adherence to the Mediterranean diet has been reported by pregnant women that are older, of higher social class, and higher education level, and who do not smoke nor drink (*p* < 0.005). In conclusion, the diet of pregnant women from Spain departs from recommendations, medium adherence to the Mediterranean diet was maintained throughout the pregnancy and post-partum, and a decreasing consumption of healthy food from the first trimester to the post-partum period was observed. Maternal factors such as age, social class, education, and smoking influence diet quality.

## 1. Introduction

Maternal nutrition during pregnancy and lactation is important since inadequate amounts of essential nutrients can adversely affect both mother and child [1,2,3,4]. During pregnancy, the development of maternal tissues, fetal growth [5,6], and breast milk production [7] increase nutritional requirements [8].

Different national and international organizations advocate dietary improvement, such as adhering to the Mediterranean dietary pattern, which is characterized by a high content of fruits, vegetables, olive oil, legumes, dairy products, and nuts, and recommend minimal intake of red meat, animal fats, sugars, and salt [9]. Several observational and intervention studies underscore the role of the Mediterranean diet in preventing type 2 diabetes mellitus, metabolic syndrome, and obesity in adults [10,11]. The Mediterranean diet has also been associated with a decrease in the risk of preterm delivery [12], gestational diabetes [13], higher birth weight [14], and obesity development in children [15,16]. Measures that promote healthy nutrition during pregnancy are therefore essential, taking into account that the maternal dietary pattern can be associated with socio-economic, cultural and lifestyle factors [12,13,17,18]. 

Despite evidence supporting the importance of maternal nutrition, various studies reveal that few women follow adequate diets [18,19,20]. In addition, there is a lack of up-to-date data on the discrepancies between nutritional intake during pregnancy and lactation and the dietary advice provided to pregnant women from high income countries. A study conducted in Spain during the first trimester of pregnancy showed intakes of cereals, legumes, fruit, and vegetables [17] under the recommended range.

In summary, the objectives of our study are as follows: (1) To assess adherence to the Spanish dietary guidelines and the Mediterranean diet; (2) to analyze the progression of the diet; and (3) to examine possible associations between food consumption and socio-economic, cultural, and lifestyle factors during pregnancy and post-partum in a sample of healthy pregnant women from a European Mediterranean country.

## 2. Materials and Methods 

### 2.1. Population and Study Design

We conducted a longitudinal study of pregnant women, with a follow-up extending to the post-partum period. Participants were healthy pregnant women from the population included in the ECLIPSES study [21], which consists of a randomized clinical trial on iron supplementation. Women were recruited during the first prenatal visit from 12 sexual and reproductive health care services (ASSIR) of the Catalan Institute of Health (ICS) in Tarragona, Spain. The ECLIPSES trial was registered in the EU Clinical Trial Register, EUCTR-2012-005480-28 and in ClinicalTrials.gov with the identification number NCT03196882. This study was approved by the Clinical Research Ethics Committee of the Jordi Gol Institute for Primary Care Research (IDIAP) and the Pere Virgili Institute for Health Research (IISPV). Informed consent was obtained from all participants. 

**Figure 1:**
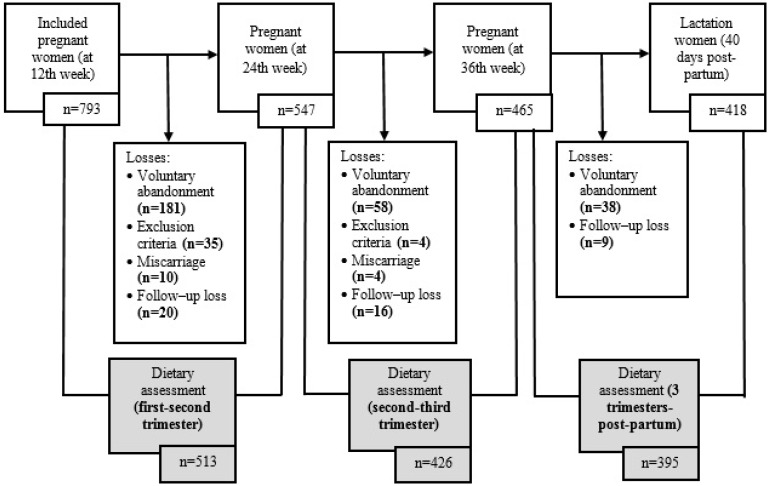
Flowchart of study participants with completed food data.

Figure 1 shows the number of participants. Of a total of 793 pregnant women included in the study at week 12 of pregnancy, 547 women provided data on week 24, 465 on week 36, and 418 at 40 days post-partum. Participant dropout was caused by voluntary abandonment (277), emergence of exclusion criteria during pregnancy (39), miscarriage (14), and lost to follow-up (45). A total of 513 participants provided data to compare the first trimester with the second, 426 to compare the second with the third, and 395 women provided data to compare the third trimester of pregnancy with the post-partum period. 

#### Selection Criteria

The inclusion criteria were as follows: Healthy adult woman older than 18 years at ≤12 weeks of gestation, able to understand the local languages (Spanish or Catalan) and the characteristics of the study, and who signed the informed consent form.

The exclusion criteria were multiple pregnancy, having taken iron supplements during the months prior to enrolment, hypersensitivity to egg protein, previous severe illness (immunosuppression), or any chronic disease that could affect nutritional development (cancer, diabetes, malabsorption, chronic hepatitis, and liver cirrhosis).

Since our aim was to describe food consumption, this study included all pregnant women in the intervention and control groups.

### 2.2. Data Collection and Processing

Midwives and nutritionists collected data on the medical history, anthropometric measurements, and lifestyle habits in the first trimester of pregnancy (at the 12th week). Nutrition was assessed at different time-points (first, second, third trimester, and post-partum) by interview using validated questionnaires.

#### 2.2.1. Medical History

The following data were collected: Maternal age, ethnicity (Spain, Latin America, and Arab), education level (primary, secondary, university studies), socio-economic level (low, middle, high), estimated date of delivery, planned pregnancy, clinical history, obstetric data, and blood pressure. The occupational status, which was classified following the Catalan classification of occupations (CCO-2011) [22], was used to calculate socio-economic level.

#### 2.2.2. Anthropometric Measures

We recorded the height (cm) and maternal weight (Kg). Based on the criteria proposed by the World Health Organization (WHO) [23], Body Mass Index (BMI) was classified as normal weight (BMI < 25 kg/m^2^) and excess weight (BMI ≥ 25 kg/m^2^). 

#### 2.2.3. Lifestyle Habits

Lifestyle habits were also recorded, including alcohol consumption and smoking. Physical activity (PA) was measured using the short version of the International Physical Activity Questionnaire (IPAQ-S) [24]. In addition, the modified Craig algorithm [25] was used to establish its adequacy level (sedentary, irregularly active, active, and very active) and women were subsequently classified into two groups: “Sedentary women”, including sedentary and irregularly active women; and “active women”, including active and very active women. 

#### 2.2.4. Food Consumption and Energy and Nutrient Intake

Eating habits were assessed through a food frequency questionnaire (FFQ) self-administered, previously validated in our population [26]. Participants reported usual food consumption retrospectively at week 12, 24, and 36 of pregnancy and at 40 days post-partum. The FFQ was explained by specialized midwives and subsequently, reviewed, entered the food data into a database and analyzed this information by nutritionists. 

The FFQ consisted of 45 items classified into 12 food groups: 1.—read and processed meat, 2.—poultry, fish, and eggs, 3.—fruits (fruit, preserved fruit), 4.—vegetables (salads and vegetables), 5.—dairy products, 6.—salted cereals (breakfast cereals, bread, pasta, and rice), 7.—sweet cereals (biscuits, pastries), 8.—legumes, 9.—nuts, 10.—sweets (sugar and chocolates), 11.—sweetened beverages, 12.—alcoholic drinks. The average ration of these groups was obtained.

The average consumption rations were compared with the dietary guidelines of the Sociedad Española de Nutrición Comunitaria (SENC) [9]. 

The consumption in grams per day of each item was calculated by applying the average ration of consumption usually performed in our population according to previous data obtained in the consumption surveys conducted by the research group [26]. For example: Milk (220 g), salad (100 g), legumes (60 g), eggs (55 g), meat (150 g), fish (150 g), fruit (100 g), among others.

#### 2.2.5. Adherence to a Relative Mediterranean Diet (rMED Score)

A scale indicating the women’s degree of adherence to the Mediterranean diet was evaluated using an rMED score, a variation of the original Mediterranean diet score [27,28] based on the intake of 9 components of this diet [29]. In order to calculate the score, each rMED component (apart from alcohol) was expressed in grams per 1000 kcal/day (to express intake as energy density) and was divided by tertiles of dietary intake. Each tertile was assigned a value of 0, 1, and 2 points. Out of the 9 components of the rMED, 6 scored positively (adherence to the Mediterranean diet): Fruit (including nuts and seeds but excluding fruit juices), vegetables (salad and vegetables), legumes, cereals (including whole-grain and refined flour, pasta, rice, other grains, and bread), fresh fish (including seafood), and olive oil. The scoring was reversed for 2 components (non-adherence to the Mediterranean diet): Total meat (including processed meat) and dairy products (milk, yogurt, cheese, and cream desserts). Alcohol, considered as harmful during this period, was scored as a dichotomous variable: A value of 0 was assigned to women who consumed alcohol and 2 to women who did not drink alcohol. The possible scores assigned to each pregnant woman ranged from 0 points (minimal adherence) to 18 points (maximum adherence). The total rMED score was classified into three categories: (1) From 0–6 it was considered as ‘‘low’’ (very low quality diet); from 7–10 as ‘‘medium’’ (needs improvement to conform to the Mediterranean diet); and from 11–18 as ‘‘high’’ (optimal Mediterranean diet).

### 2.3. Statistical Analysis

The results are presented as percentages, mean and standard deviation, or median and interquartile range. The normality of the variables was analyzed. Since the distribution of food consumption was skewed, non-parametric tests were used. We compared the median between the first and the second trimester, the second and the third trimester, the third trimester to post-partum, and the mean of the three trimesters to post-partum, using the Wilcoxon signed-rank test. To analyze the differences in food consumption according to maternal factors, we used the Mann-Whitney *U* test for variables with two categories (maternal alcohol consumption, maternal smoking, and planned pregnancy), and the Kruskal-Wallis test for variables with more than two categories (socio-economic level, maternal education level, country of origin, age). The level of significance was set at *p* < 0.05 in all tests. SPSS version 20.0 was used for statistical analysis.

## 3. Results

### 3.1. Participants’ Characteristics

General characteristics of pregnant women in the first trimester are shown in Table 1. A total of 793 women (mean age 30.5 ± 4.9 years) were enrolled in the study. Up to 40.9% women completed at least secondary studies, and 68.3% were middle-class. About 15% women smoked and consumed alcohol during pregnancy, 36.6% were overweight, and 92.9% were sedentary. 

### 3.2. Food Consumption 

Table 2 shows food consumption in the first trimester. For the whole population, mean and median intakes (in servings/d and g/d) were outside the recommended range for all food groups. The total rMED score obtained was medium.

### 3.3. Food Consumption throughout Pregnancy and Post-Partum 

Table 3 shows the results of the median daily food consumption and Mediterranean diet adherence during pregnancy and post-partum. When comparing the first versus the second trimester, we observed a significantly lower intake of fruit and salted cereals and a higher intake of dairy products, sweets, and sweetened beverages during the second trimester. Comparing the second with the third trimester, we observed a significantly lower intake of salted cereals in the third trimester. When comparing the third trimester versus the post-partum period, we observed a significantly lower intake of fruits and dairy products and a higher intake of salted cereals and legumes during the post-partum period. When the mean daily food consumption of the three trimesters was compared against the post-partum, a significantly lower intake of fruits, vegetables, salted, and sweet cereals was observed during the post-partum period. 

### 3.4. Influence of Maternal Factors on Food Consumption

As shown in the first trimester of pregnancy (Table 4), women of higher social class consumed a significantly greater amount of vegetables, salted cereals, and nuts; more educated women had a significantly higher intake of vegetables and nuts and presented higher adherence to the Mediterranean diet; Spanish women had a significantly higher intake of red and processed meat, vegetables, sweets, and sweetened beverages and lower intake of fruits and salted cereals compared with women of foreign origin; older women consumed a significantly greater amount of vegetables and nuts and together with women 25 to 30 years of age, they presented a higher adherence to the Mediterranean diet; women who consumed alcohol had a significantly higher intake of fruits, vegetables, dairy products, sweet cereals, nuts, sweets, and sweetened beverages, but presented a lower score in adherence to the Mediterranean diet; women who smoked during pregnancy had a significantly lower and higher intake of fruits and sweetened beverages, respectively; and women who planned their pregnancy had a significantly higher intake of vegetables and legumes.

Although the results obtained after the first trimester were similar to the first trimester of pregnancy, the following differences were detected: Women of lower social class had a significantly higher intake of sweetened beverages during the third trimester; less educated women consumed a significantly lower amount of dairy products, salted cereals, and had a higher intake of sweetened beverages during the post-partum period; Arab women presented a significantly higher adherence to the Mediterranean diet during the post-partum period; older women presented a significantly higher adherence to the Mediterranean diet during the second trimester; finally, women who smoked during pregnancy consumed a significantly greater amount of red and processed meat, sweet cereals, legumes, and sweetened beverages during the second trimester.

## 4. Discussion

This study describes the food consumption and adherence to the Mediterranean diet pattern during pregnancy and the post-partum period of women from a high income country with sufficient food availability. The results show discrepancies between the actual diets of participants and the national food recommendations and medium adherence to the Mediterranean diet, mainly due to low consumption of healthy food and high consumption of sugary food and drinks and red and processed meat. The evolution of food consumption was unfavorable, in particular regarding a decrease in fruit, vegetable, and salted cereals intake during the post-partum period. Notably, poor diet quality was associated with younger age, lower social class and education level, smoking, and alcohol consumption. In contrast, women who are older, of a higher social class, and better educated, and women who do not smoke and do not drink have a higher quality diet and better adherence to the Mediterranean diet. 

### 4.1. Adherence to the Spanish Dietary Guidelines and to the Mediterranean Diet Pattern

There is enough evidence about the impact of the diet of pregnant women on the health of the mother and the child’s development [30]. However, despite sufficient availability, consumption of fruit, vegetables, salted cereals, legumes, nuts, dairy products, poultry, fish, and eggs during pregnancy was below recommendations. Previous studies conducted in our region show similar findings [17,18,20,31,32]. For instance, a cross-sectional study conducted in a cohort of Mediterranean Spanish pregnant women observed that over fifty percent did not meet the recommendations for cereals, legumes, milk, and dairy products [17]. In a sample of 13,845 women from various Spanish regions, Cuervo et al. [20] found that the consumption of high protein food, dairy products, cereals, and vegetables during pregnancy was lower than recommended. In contrast and in agreement with our results, they found an excess consumption of sausages, buns, and pastries. It should be noted that while the guidelines recommend to only eat these types of food (red and processed meat, sweets, sweet cereals, and sugary drinks) occasionally, our results show that they were consumed frequently during pregnancy. Another study conducted in our region identified a food pattern rich in sugary drinks and food from preconception to six months of gestation [32]. Other studies conducted in high income countries have observed similar food consumption patterns. In Canada, Savard et al. [33] found a low consumption of fruit, vegetables, and whole grain products and a high consumption of saturated fat in a sample of 79 women. In an Australian cohort of 1570 women, Lee at al. [34] reported that more than sixty percent consumed less than the DRI of vegetables, grains, meat, and meat alternatives.

The Mediterranean diet during pregnancy might have beneficial effects on the newborn and on children’s health [29,35,36]. Our pregnant women showed a medium adherence to the Mediterranean diet in the assessment of diet quality. A better adherence to the Mediterranean diet has been associated with appropriate weight gain, which could protect against overweight and obesity during pregnancy [37]. We consider this aspect relevant since 36.6% of women in our sample had excess weight in the first trimester.

Globally, unhealthy dietary habits in pregnant women are associated with negative health consequences and increased risk of nutritional deficiencies. For instance, deficient calcium intake increases the risk of preeclampsia, affects bone health, and is associated with restricted fetal growth and low birth weight [30]. Low consumption of vegetables, fruits, nuts, legumes, and whole cereals contribute to insufficient intake of fiber and folic acid. Adequate fiber intake can improve and prevent constipation, reduce blood cholesterol, and control blood glucose to prevent gestational diabetes [30]. The adequate intake of folic acid is essential during the embryonic and fetal stages of pregnancy and its deficiency increases the risk of preeclampsia and fetal anomalies [30]. On the other hand, over-consumption of unhealthy food may also impact on health [38]. For instance, sweetened beverages are associated with low diet quality and a high caloric intake [39], and the excessive consumption of free sugars has been associated with gestational diabetes [40].

### 4.2. Diet Changes during Pregnancy and Post-Partum

The analysis of dietary changes during the three trimesters of pregnancy and the post-partum period has revealed unfavorable trends. The results show that adherence to the Mediterranean diet remains unchanged during pregnancy. However, the consumption of healthy food decreases progressively until the post-partum period. Except for an increase of dairy products, there is a progressive decrease in the intake of fruit, vegetables, and salted cereals. In contrast, the intake of unhealthy food presents an upward trend, mainly from the first to the second trimester, which translate into a higher consumption of sweets and sweetened beverages. The consumption of unhealthy food might displace recommended food and increase the risk of inadequate nutrient intake [6,30]. The increase of sweet food and beverages from the first to the second trimester probably responds to cravings, since changes in satiety and food choices are frequent during pregnancy. Interestingly, Savard et al. [33] found that food cravings in the second trimester decreased by 1.6 points the Healthy Eating Index score. Furthermore, the decrease of salted cereals throughout the three trimesters might cause an insufficient intake of carbohydrates, which can derive into an energy intake below the caloric demand of gestation. On the other hand, this decline of intake of salted cereals might respond to the general health advice to control weight gain during pregnancy. 

To our knowledge, there are few studies which provide a longitudinal analysis on food consumption and diet quality during gestation [6,33,41,42,43]. Savard et al. [33] did not observe a change on diet quality index, but they reported a significant decrease in the intake of fruit, vegetables, unsaturated, and saturated fat from the first to the third trimester and an increase in the intake of milk and dairy products in the third trimester. Rifas-Shiman et al. [43] did not find significant changes in food consumption from the first to the second trimester, despite reporting a slight increase of dairy products, red and processed meat, and saturated fat. In contrast, other studies report changes in food consumption during pregnancy. For instance, an increased intake of fruit, vegetables, and milk was observed across gestation in a sample of 80 Spanish pregnant women [6]. In this study, the favorable changes in food consumption as pregnancy progresses could be explained by the sample of participants, which belonged to a high socio-economic level and were very collaborative, and also by the methodology used to assess food consumption. 

In agreement with our results, Looman et al. [42] found a significant change on diet quality from early pregnancy to the second trimester in Dutch pregnant women. In Australia, Moran et al. [41] showed a decrease in diet quality across gestation only in a group of obese pregnant women.

To date, few studies have been published on dietary intake from pregnancy to post-partum [6,20,32,41,44]. In this research, we evaluated the immediate post-partum (up to 40 days after delivery) and observed the same progression described during pregnancy, with no change regarding adherence to the Mediterranean diet, but an increasing lower consumption of foods associated with a healthy diet. This trend was also observed in the same population at 26 weeks post-partum (with a decreased intake of fruits, vegetables, cereals, and oils) [6] and in a greater sample of breastfeeding women at six months post-partum (with a decrease in the intake of cereals, vegetables, fruits, and dairy products) [20]. A study conducted in 32 healthy German women at six weeks post-partum coincides with our results with regard to a decrease in carbohydrate intake [44]. Another study found a decrease in diet quality and a reduction in the consumption of milk, meat, and oil from pregnancy to four months post-partum in a group of Australian pregnant women with obesity [41].

It is possible that the dietary restriction observed after delivery is caused by the desire to shed excess pregnancy weight. However, since over 70% of women in our study chose to breastfeed, it is important to remember that energy and nutrient intake recommendations increase during lactation. 

In summary, we observed a progressive decline in the consumption of healthy food from pregnancy to post-partum and an increased risk of inadequate intake of vitamins and minerals in the post-partum period [6,45]. However, the women in our sample maintained medium level of adherence to the Mediterranean diet pattern throughout the study. 

### 4.3. Association with Socio-Economic and Cultural Characteristics and Lifestyles 

Several barriers to a healthy diet in pregnancy have been identified. For instance, lack of nutrition knowledge [33] and limited nutrition education from the health care provider [46]. Pregnant women reported that dietary information from different sources seemed contradictory and that the information provided by the healthcare provider was insufficient and inaccurate [46,47,48]. In the public health system of our region, current clinical guidelines for pregnant women [49] include dietary advice, which should be provided by midwives with their limited allocated time. There are no nutritionists in charge of promoting and monitoring the diet during pregnancy. Together with current evidence, our findings highlight the need for intensive nutritional education and dietary advice at the start of the pregnancy by a nutrition expert to contribute to adequate nutrition and health. 

The factors that influence the diet need to be considered to ensure that nutritional advice reaches all pregnant women. Our study found that a higher socio-economic class, education level, age, and not smoking nor drinking were associated with a higher reported adherence to the Mediterranean diet. Age is considered one of the most determinant factors of diet [12,17,18,32,33,50,51,52] and older age was associated with a higher level of education [19]. The results of this research show that older women consume a greater amount of vegetables, salted cereals, nuts, and legumes, and less red and processed meat and sweet cereals. Consumption of sweet cereals is higher in women of lower social class and those with only primary schooling. Similar findings were obtained by other researchers [17,18,19,34,51,52]. For instance, Rodríguez-Bernal et al. [17] showed that less education and younger age were associated with lower consumption of vegetables and high protein foods, respectively, lower intake of n-3 fatty acids and higher intakes of trans fatty acids. In a sample of 567 Swedish pregnant women, Stravik et al. [19] found that a higher consumption of fruits, vegetables, whole cereals, and fish was associated with higher educational level and older age. Lee et al. [34] presented an association between higher level of education and adequate food intake. Other studies found that older age and higher level of education was related with a healthier or prudent dietary pattern [12,51] and better diet quality [52]. Savard et al. [33] showed higher quality diets in the third trimester in women over 28 years of age and higher education level. In this cohort, older women reported a higher adherence to the Mediterranean diet in the first and second trimester. In addition, the association between higher education level and older age with higher consumption of vegetables and nuts remained constant throughout pregnancy. At post-partum, a lower education level was associated with a lower consumption of dairy products, salted cereals, and a higher intake of sweetened beverages.

In relation to the country of origin, our study revealed that Spanish pregnant women consume more vegetables than Latin American and Arab women, but less salted cereals and fruits and more red and processed meat, sweets, and sweetened beverages. In a study conducted in the island of Menorca (Balearic Islands), Ferré et al. [7] associated the healthy dietary pattern with foreign-born women and also with women from continental Spain. Another study conducted in Spain reported that women ate less fruit and carbohydrates and higher amounts of protein than foreign-born women [17]. Differences in results might be attributed to the wide percentage range of foreign-born women (from 3 to 20%), the diversity of countries of origin and other migrant conditions that affect food choices. Taking into account modifiable lifestyle maternal factors, we observed that pregnant women that consumed alcohol had a lower adherence to the Mediterranean diet, but a higher intake of vegetables and fruits and a lower intake of red and processed meat, sweet food, and sugary drinks than pregnant women that do not consume alcohol. In contrast, a study by Coathup et al. [53] associated a greater intake of processed foods with heavier alcohol consumption. However, few studies address the influence of alcohol consumption on diet quality. It is possible that the pregnant population is not expected to have a high consumption of alcohol and they are not asked about it in regular pregnancy visits. Interestingly, we found that when assessed through self-reporting food frequency questionnaires, 15% of women admit to drinking alcohol, whereas when the health practitioner asks about drinking, only 4% admit to consuming alcohol (data not shown). On the other hand, we found that women who smoke during the first trimester consume less fruit and more sweetened beverages throughout the pregnancy and post-partum periods, and a greater amount of red and processed meat, sweet cereals, and legumes in the second trimester. Similarly, Stravick et al. [19] found an association between smoking and low consumption of fruits. Other studies associated sweetened beverages and sugar dietary pattern with smoking [7,32]. In conclusion, healthy lifestyle behaviors are associated with higher diet quality, and therefore health programs for pregnant women should focus on modifiable factors and consider interventions for women who smoke and who drink alcohol. 

Remarkably, while a recent review reported that age and education are the main factors associated with low quality diet, there is still a lack of evidence on other factors like ethnicity, planned pregnancy, maternal illness, lifestyle, and toxic habits like smoking and drinking alcohol [50]. Our findings add new data to scientific evidence and show maternal characteristic and modifiable factors that should be considered to emphasize dietary recommendations. We underscore the importance of assessing the diet during gestation and the need for further research on the effect of maternal and environmental factors on food consumption.

In general, these findings show that diet in pregnancy has not improved despite evidence on the importance of nutrition in mother and child. Consequently, we believe that dietary interventions should be provided by nutritional experts to optimize the diet of mothers during pregnancy and after delivery to maintain a healthy weight and to prevent negative health outcomes.

### 4.4. Strengths and Limitations

This study furthers the understanding on food consumption, adherence to recommendations, and to the Mediterranean diet across pregnancy and the post-partum period in our region. It also identifies the maternal factors that influence diet. One of the major strengths of the study is the prospective design and the large sample size. Moreover, we use a semiquantitative FFQ validated in our population [26] which is easy to administer. Our study also has some limitations. Firstly, complete dietary data decreased from pregnancy to post-partum, with only 49.8% of participants providing all the information. Moreover, due to the design of the clinical trial, women who took iron supplementation were excluded. Secondly, we were unable to evaluate other environmental and maternal factors that might impact on food consumption during pregnancy [50]. Finally, our results show that pregnant women must be accurately advised on the need of an adequate diet during and after pregnancy, promoting the Mediterranean diet, the healthy diet model typical of our region. Moreover, the design of the interventional education programs should take into account factors that affect food consumption, focusing on women that are younger, less educated, and from lower socio-economic strata, and on other modifiable factors such as smoking and drinking. Further research is needed for an in-depth assessment of dietary intake and personal and environmental factors involved. 

## 5. Conclusions

Consumption of food in pregnant women from Spain does not meet recommendations. However, this population showed medium adherence to the Mediterranean diet pattern that did not change throughout gestation and post-partum. The consumption of healthy foods evolved unfavorably throughout pregnancy and the post-partum period. Higher levels of adherence to the Mediterranean diet were observed in pregnant women that were older, had a higher education level, and who did not drink alcohol. These results should be taken into account when implementing nutritional intervention programs, which should focus on a healthy diet such as the Mediterranean diet for pregnant and lactating women, in order to prevent nutritional deficiencies that might adversely impact the health of the mother and the new-born. Further research is needed to identify the personal and environmental factors that contribute to a healthy diet. 

## Figures and Tables

**Table 1:** Sociodemographic and lifestyle characteristics of pregnant women in the first trimester of pregnancy.

General Characteristics		*n* = 793
**Maternal age (years)**		30.5 (4.9)
**Country of origin, Spain (%)**		84.1 (667)
**Planned pregnancy (%)**		80.3 (611)
**Primipara (%)**		37.5 (297)
**Gestational age (weeks)**		39.7 (0.9)
**Type of delivery (%)**	Normal vaginal	80 (634)
	Caesarean	20 (159)
**Maternal educational level (%)**	Primary studies	31.4 (249)
	Secondary studies	40.9 (324)
	University studies	27.7 (220)
**Social class (%)**	Low	16.0 (127)
	Middle	68.3 (542)
	High	15.6 (124)
**Maternal smoking (%)**		15.3 (121)
**Maternal alcohol consumption (%)**		15.4 (122)
**Weight (kg)**		65.1 (11.9)
**Height (m)**		1.61 (0.1)
**BMI (kg/m^2^)**		24.8 (4.5)
**BMI categories (%)**	Normal weight	63.4 (503)
	Excess weight	36.6 (290)
**Physical activity categories (%)**	Active	7.1 (56)
	Sedentary	92.9 (737)
**Type of feeding at 40 days (%)**	Breastfeeding	72.5 (575)
	Mixed feeding	9.5 (75)
	Infant formula	18 (143)

Values are expressed in mean (SD) or % of pregnant women in each category. BMI = Body Mass Index.

**Table 2:** Food consumption in pregnant women in the first trimester of pregnancy.

				*n* = 793	
Food Group	Recommended * (Servings)	Servings/d		g/d	
		Mean (SD)	Median (IQR)	Mean (SD)	Median (IQR)
**Red and processed meat**	Occasionally	0.95 (0.5)	1 (0.7)	57.1 (31.8)	51.7 (43.1)
**Poultry, fish, and eggs**	2 per day	1.1 (0.5)	1.1 (0.6)	97.4 (44.6)	98.6 (57.1)
**Fruits**	3–4 per day	1.7 (1.0)	1.7 (1.3)	229.8 (141.9)	228.6 (195.6)
**Vegetables**	2–3 per day	1.2 (0.7)	1.1 (0.9)	74.8 (44.8)	71.4 (48.21)
**Dairy products**	3–4 per day	2.2 (1.2)	2.2 (1.3)	309.3 (175.7)	317.1 (184.2)
**Salted cereals ^‡^**	4–5 per day	1.8 (0.8)	1.8 (0.9)	124.5 (54.4)	122.1 (60.83)
**Sweet cereals ^†^**	Occasionally	1.0 (0.8)	0.9 (0.9)	33.9 (28.3)	30.4 (30.7)
**Legumes**	2–4 per week	0.2 (0.2)	0.2 (0.1)	14.6 (11.4)	15.2 (8.6)
**Nuts**	3–7 per week	0.2 (0.2)	0.1 (0.3)	2.8 (3.5)	2.0 (4.3)
**Sweets**	Occasionally	0.3 (0.4)	0.2 (0.4)	5.5 (6.6)	3.6 (6.0)
**Sweetened beverages**	Occasionally	0.2 (0.3)	0.1 (0.3)	49.3 (64.3)	28.6 (57.1)
**Alcoholic drinks**	None	0.02 (0.1)	0.0 (0.0)	2.5 (12.1)	0.0 (0.0)
**rMED Score •**	-	9.5 (2.6)	9.8 (3.0)	-	-

Values are expressed in mean (SD) and median (interquartile range) * Food-based dietary guidelines of the Spanish Society of Community Nutrition, 2019; ‡ Salted cereals include flour, pasta, rice, bread, and potatoes. † Sweet cereals include sweetened breakfast cereals, biscuits, and baked goods. • Relative Mediterranean Diet Score.

**Table 3:** Food consumption and adherence to the Mediterranean diet throughout pregnancy and post-partum.

	Comparison First Trimester Versus Second Trimester			Comparison Second Trimester Versus Third Trimester		Comparison Third Trimester Versus Post-Partum		Comparison Mean Three Trimesters Versus Post-Partum		
	First Trimester ^a^ *n* = 513	Second Trimester ^b^	*p*-Value between ^(a-b)^	Third TRIMESTER ^c^	*p*-Value between ^(b-c)^	Post-Partum ^d^	*p*-Value between ^(c-d)^	Mean Three Trimesters ^e^	Post-Partum ^d^	*p*-Value between ^(d-e)^
	*n* = 513	*n* = 513		*n* = 426		*n* = 395		*n* = 395	*n* = 395	
	Median (IQR)	Median (IQR)		Median (IQR)		Median (IQR)		Median (IQR)	Median (IQR)	
**Red and processed meat**	58.6 (37.1)	60.0 (38.4)	0.306	58.6 (37.2)	0.315	58.6 (35.0)	0.566	59.2 (34.0)	58.6 (35.0)	0.444
**Poultry, fish, and eggs**	96.8 (60.4)	100.7 (53.0)	0.577	92.1 (55.9)	0.311	96.9 (50.7)	0.752	99.5 (51.0)	96.9 (50.7)	0.989
**Fruits**	228.6 (208.9)	214.3 (185.7)	0.045	209.8 (178)	0.088	189.0 (189.3)	0.003	221.4 (159.5)	189.0 (189.3)	<0.001
**Vegetables** **Dairy products**	71.4 (50.1)	72.9 (51.4)	0.881	72.9 (48.9)	0.252	71.4 (45.4)	0.873	75.7 (46.9)	71.4 (45.4)	0.003
**Dairy products**	319.3 (163.9)	337.1 (141.4)	<0.001	337.1 (141.2)	0.789	319.3 (137.1)	0.022	332.1 (135.6)	319.3 (137.1)	0.242
**Salted cereals ^‡^** **Sweet cereals ^†^**	121.1 (63.6)			110.7 (59.8)	0.110	116.4 (11			116.4 (11)	0.040.
		116.4 (59.8)	<0.001	110.7 (59.8)	0.011	116.4 (58.5)	0.049	117.4 (27.0)	116.4 (58.5)	0.047
**Sweet cereals ^†^**	30.0 (34.4)									
		30.9 (30.6)	0.764	30.0 (30.8)	0.449	29.3 (28.6)	0.710	31.2 (26.9)	29.3 (28.6)	0.033
**Legumes**	17.1 (8.6)	17.1 (8.6)	0.398	12.9 (8.6)	0.201	17.1 (8.6)	0.042	14.3 (11.4)	17.1 (8.6)	0.653
**Nuts**	2.0 (4.3)	2.1 (4.3)	0.947	1.8 (4.3)	0.280	2.1 (4.3)	0.123	2.1 (3.6)	2.1 (4.3)	0.739
**Sweets**	3.6 (7.6)	4.3 (7.6)	0.006	4.3 (7.1)	0.418	3.9 (7.4)	0.409	4.6 (6.6)	3.9 (7.4)	0.996
**Sweetened beverages**	28.6 (85.7)	32.3 (74.7)	0.004	32.6 (73.8)	0.868	28.6 (85.7)	0.629	37.5 (71.4)	28.6 (85.7)	0.579
**Alcoholic drinks**	0.0 (0.0)	0.0 (0.0)	0.682	0.0 (0)	0.515	0.0 (0.0)	<0.001	0.0 (0.0)	0.0 (0.0)	<0.001
**rMED Score •**	10.0 (4.0)	10.0 (4.0)	0.833	10.0 (4)	0.819	10.0 (4.0)	0.288	10.0 (3.0)	10.0 (4.0)	0.110

Values are expressed in median (interquartile range). ^‡^ Salted cereals include flour, pasta, rice, bread, and potatoes. ^†^ Sweet cereals include sweetened breakfast cereals, biscuits, and baked goods. • Relative Mediterranean Diet Score. ^a^ First trimester. ^b^ Second trimester. ^c^ Third trimester. ^d^ Post-Partum. ^e^ Mean three trimester.

**Table 4:** Intake of food groups (g/d) and relative Mediterranean Diet Score during the first trimester of pregnancy according to maternal factors.

Food Group (g/d)	Socio-economic Level				Maternal Educational Level				Country of Origin				
	Low *n* = 107	Medium *n* = 440	High *n* = 110	*p*-Value	Primary *n* = 206	Secondary *n* = 252	University *n* = 199	*p*-Value	Spain *n* = 544	Latin America *n* = 61	Arab *n* = 52	*p*-Value	
	Median (IQR)	Median (IQR)	Median (IQR)		Median (IQR)	Median (IQR)	Median (IQR)		Median (IQR)	Median (IQR)	Median (IQR)		
Red and processed meat	55.0 (50.9)	52.7 (43.1)	52.1 (41.6)	0.734	55.0 (43.0)	55.0 (47.2)	51.4 (38.8)	0.308	55.0 (42.9)	50.0 (54.7)	40.0 (42.1)	0.011	
Poultry, fish, and eggs	102.3 (61.9)	98.5(54.5)	102.3 (52.3)	0.423	101.1 (53.9)	100.7 (56.0)	102.3 (48.6)	0.661	101.2 (53.6)	101.4 (60.2)	102.3 (56.0)	0.564	
Fruits	234.4 (185.7)	228.6 (185.1)	234.4 (180.4)	0.904	227.6 (228.6)	228.6 (176.8)	234.4 (174.3)	0.518	220.7 (177.4)	285.7 (133.8)	248.2 (253.6)	0.003	
Vegetables	64.3 (44.5)	70.7 (41.4)	79.3 (51.8)	<0.001	63.6 (41.9)	70.0 (42.5)	78.6 (45.0)	<0.001	75.7 (48.6)	65.7 (37.5)	60.7 (52.0)	0.008	
Dairy products	333.6 (157.9)	319.6 (204.5)	322.5 (152.5)	0.745	308.2 (227.1)	330.0 (156.6)	333.6 (167.9)	0.417	322.1 (180.3)	333.3 (160.3)	321.5 (227.5)	0.748	
Salted cereals ^‡^	121.3 (81.1)	119.5 (58.7)	124.3 (39.5)	0.016	124.3 (73.9)	124.3 (55.4)	119.6 (52.1)	0.174	120.7 (54.1)	140.7 (106.4)	123.4 (81.6)	0.009	
Sweet cereals ^†^	35.0 (37.9)	32.1 (29.4)	29.5 (24.1)	0.166	32.4 (38.1)	35 (29.1)	26.4 (25.5)	0.063	31.3 (27.5)	35.9 (43.0)	35.0 (37.4)	0.947	
Legumes	15.2 (17.1)	15.1 (8.6)	15.2 (8.6)	0.059	15.2 (9.6)	15.2 (8.6)	15.2 (8.6)	0.688	15.2 (8.6)	15.2 (21.6)	12.9 (18.2)	0.959	
Nuts	0.5 (2.9)	2.1 (4.3)	2.9 (5.9)	<0.001	1.0 (2.9)	2.1 (4.3)	2.1 (3.8)	<0.001	2.1 (4.3)	0.5 (2.9)	2.3 (6.4)	0.043	
Sweets	2.9 (7.6)	3.6 (6.0)	3.9 (5.7)	0.933	3.1 (5.7)	3.8 (7.1)	3.6 (5.7)	0.642	3.6 (6.0)	2.1 (6.0)	2.9 (5.9)	0.013	
Sweetened beverages	28.6 (57.1)	28.6 (85.7)	28.6 (57.1)	0.432	28.6 (85.7)	28.6 (57.1)	28.6 (57.1)	0.324	28.6 (57.1)	14.3 (56.0)	20.5 (57.1)	0.048	
Alcoholic drinks	0.0	0.0	0.0	0.825	0.0	0.0	0.0	0.643	0.0	0.0	0.0	0.278	
rMED Score •	9.8 (3.0)	9.8 (3.0)	10.0 (3.0)	0.065	9.8 (4.0)	9.8 (3.8)	10.0 (4.0)	0.012	9.8 (3.0)	9.8 (4.0)	10.0 (3.0)	0.070	
**Food group (g/d)**	**Age (years)**				**Maternal alcohol consumption**			**Maternal smoking**			**Planned pregnancy**		
	**<25** ***n* = 85**	**25–29** ***n* = 168**	**≥30** ***n* = 404**	***p*-value**	**No** ***n* = 671**	**Yes** ***n* = 122**	***p*-value**	**No** ***n* = 546**	**Yes** ***n* = 111**	***p*-value**	**No** ***n* = 129**	**Yes** ***n* = 520**	***p*-value**
	**Median (IQR)**	**Median (IQR)**	**Median (IQR)**		**Median (IQR)**	**Median (IQR)**		**Median (IQR)**	**Median (IQR)**		**Median (IQR)**	**Median (IQR)**	
Red and processed meat	46.2 (42.6)	56.8 (37.5)	50.7 (41.6)	0.014	55.7 (40.0)	27.0 (32.0)	<0.001	51.6 (43.3)	55.7 (42.9)	0.292	50.7 (45.8)	54.6 (41.6)	0.535
Poultry, fish, and eggs	92.9 (59.5)	99.3 (57.8)	102.3 (52.6)	0.505	93.6 (58.8)	102.3 (9.8)	0.053	101,43 (51.3)	101.4 (69.6)	0.361	101.9 (65.4)	101.4 (52)	0.818
Fruits	214.3 (221.4)	228.6 (197.0)	234.4 (173.9)	0.510	214.3 (200.0)	234.4 (37.1)	0.002	234.4 (179.3)	185.7 (214.3)	0.005	233.3 (189.8)	228.6 (185.7)	0.433
Vegetables	51.4 (50.0)	67.1 (49.0)	78.6 (44.3)	<0.001	65.7 (51.4)	78.6 (3.5)	<0.001	74.3 (45.5)	64.3 (51.4)	0.070	67.1 (48.4)	74.3 (47.1)	0.018
Dairy products	297.9 (230.4)	308.6 (207.9)	335.1 (151.9)	0.140	308.6 (217.1)	335.1 (16.6)	0.045	323 (173.3)	320 (222.1)	0.643	315.7 (183)	322.1 (178.7)	0.408
Salted cereals ^‡^	124.3 (88.0)	124.3 (62.2)	120.9 (54.3)	0.141	117.9 (70.6)	124.3 (4.0)	0.183	123.4 (60.1)	117.9 (68.6)	0.164	124.3 (71.5)	120.7 (57.4)	0.179
Sweet cereals ^†^	35.0 (47.1)	32.9 (33.2)	30.1 (28.8)	0.068	27.1 (35.0)	35.9 (8.0)	0.003	32.1 (28.2)	30 (33.8)	0.600	30.4 (33.8)	31.6 (29.2)	0.700
Legumes	15.2 (20.4)	15.2 (8.6)	15.2 (8.6)	0.957	8.6 (8.6)	15.2 (6.6)	0.368	15.2 (8.6)	17.1 (8.6)	0.618	8.6 (12.9)	15.2 (8.6)	0.031
Nuts	1.0 (2.9)	1.1 (4.3)	2.1 (4.3)	0.003	1.1 (4.3)	2.9 (0.7)	<0.001	2.1 (4.3)	1.1 (2.9)	0.102	1.1 (2.9)	2.1 (4.3)	0.058
Sweets	3.6 (6.4)	3.6 (7.8)	3.6 (5.7)	0.678	3.0 (6.6)	6.1 (2.6)	<0.001	3.6 (5.9)	3.6 (8.5)	0.884	2.9 (5.6)	3.6 (6.4)	0.135
Sweetened beverages	28.6 (85.7)	28.6 (85.7)	28.6 (57.1)	0.918	26.7 (57.1)	56.0 (27.4)	<0.001	28.6 (57.1)	56 (114.3)	0.044	28.6 (78.6)	28.6 (57.1)	0.867
Alcoholic drinks	0.0	0.0	0.0	0.531	0.0	6.3 (9.8)	<0.001	0 (0)	0 (0)	0.500	0 (0)	0 (0)	0.650
rMED Score •	9.0 (3.5)	9.9 (3.0)	9.8 (3.0)	0.026	10.0 (4.0)	9.8 (1.8)	0.004	9.8 (3)	9 (4.7)	0.079	9.8 (3)	9.8 (3)	0.172

Values are expressed in median (interquartile range); Food-based dietary guidelines of the Spanish Society of Community Nutrition, 2019; ‡ Salted cereals include flour, pasta, rice, bread and potatoes. † Sweet cereals include sweetened breakfast cereals, biscuits and baked goods. • Relative Mediterranean Diet Score.
